# Association between Tai Chi Yuttari Exercise and Longevity and Prevention of Long-Term Care Need: Survival Analysis in Kitakata City, Japan

**DOI:** 10.3390/ijerph20043472

**Published:** 2023-02-16

**Authors:** Nobuaki Moriyama, Tomomi Kuga, Tetsuzo Oshima, Kumiko Sato, Miho Kurita, Seiji Yasumura

**Affiliations:** 1 Department of Public Health, Fukushima Medical University School of Medicine, Fukushima 960-1295, Japan; 2 Health and Welfare Department, Elderly Welfare Division, Kitakata 966-8601, Japan

**Keywords:** Tai Chi Yuttari exercise, longevity, long-term care need, older people

## Abstract

This study examined whether participation in Tai Chi Yuttari exercise is associated with a delay in the death and new certification for long-term care need of older adults. Individuals who participated in Tai Chi Yuttari exercise classes in 2011–2015 (participation group) were compared with individuals from the Basic Resident Register of Kitakata City (non-participation group). Death and new certification for long-term care need were selected to evaluate the effectiveness of participation in Tai Chi Yuttari exercise classes. The periods from the start date of the observation to each person’s date of occurrence of events were calculated. The Kaplan–Meier method and log-rank test were used to compare survival curves between the groups. A total of 105 and 202 individuals in the participation and non-participation groups, respectively, were observed. Survival duration (χ^2^ = 8.782, *p* = 0.003) and the period before receiving certification for long-term care (χ^2^ = 5.354, *p* = 0.021) were longer in the participation group than in the non-participation group. In the stratified analysis by sex, survival duration was longer in the participation group in men only (χ^2^ = 7.875, *p* = 0.005). Participation in Tai Chi Yuttari exercise might be effective in delaying death, especially in men, and new certification for long-term care.

## 1. Introduction

Many countries globally are experiencing an unprecedented and rapid rise in the number of older people [1]. Aging is a well-known risk factor for multiple non-communicable diseases (NCDs) [2], which creates a heavy economic burden in both developing and developed countries [3,4] and threatens the financial sustainability of all healthcare systems. Furthermore, a recent study reported that old-age life expectancy reflects the health and well-being of the population, and NCD-related mortality is a key factor affecting life expectancy in developed countries [4]. 

Especially in Japan, a rapid increase has occurred in the percentage of those aged ≥65 years. In 2017, it rose to 27.7%, and the percentage of households with individuals aged ≥65 years was 48.4% in 2016 [5]. Life expectancy at birth, which is one of the most frequently used health status indicators [6], increased rapidly in the 1950s and early 1960s as a result of decreased mortality rates for communicable diseases in children and young adults [7]. Currently, addressing modifiable risk factors, including physical inactivity [8], is thought to be a key strategy for preventing NCDs [9] and subsequently improving life expectancy.

In addition, improving longevity without aid from others is also an important goal in this super-aged society. Declining function caused by aging is inevitable for all individuals, and age is a major component of the scales for predicting care needs [10]. To support older people who are dependent on others in their daily functions, Japan has provided a long-term care (LTC) insurance program in which certified individuals can choose and receive integrated health, medicine, and welfare services from diverse agents since 2000 [11]. As the number of older people increases, the number of older people with LTC certification continues to increase [12]. In Japan, the main conditions for needing LTC are cerebrovascular disease (17.2%), dementia (16.4%), age-related frailty (13.9%), and falls and fractures (12.2%) [13]. To reduce the risk of these diseases or conditions, efficient strategies to prevent LTC need should be considered.

Lifestyle is a key factor influencing longevity and functional ability in older people. Ekblom-Bak et al. [14] reported that a generally active daily life was associated with longevity in older adults, regardless of regular exercise. In addition, a systematic review examining the physical activity of healthy community-dwelling older (>65 years) adults found a consistent result of studies suggesting that regular aerobic activity is effective in the reduction in the risk of functional limitations [15]. Genetic factors and lifestyle habits are believed to influence life expectancy through the efficiency of exercise (the efficiency with which energy is converted into movement), the ability to perform activities of daily living, and the degree of independence [16]. Thus, exercise habits could influence life expectancy.

Tai Chi is a type of exercise involving systematic calisthenics, which is widely practiced in China. Tai Chi has been shown to be effective in preventing falls [17] and promoting the overall well-being of older adults [18]. Kitakata City in Fukushima Prefecture developed Tai Chi Yuttari (“Yuttari” means “relaxed” or “slowly” in Japanese) exercise classes for slightly frail older people to prevent deterioration of physical function and the need for LTC. Tai Chi Yuttari exercise focuses on the health effects of Tai Chi in the prevention of LTC, and Kitakata City started to offer Tai Chi Yuttari exercise classes in collaboration with Fukushima Medical University and Aizu Health and Welfare Office in 2007 [19]. Regarding the effectiveness of Tai Chi Yuttari exercise, Fujimoto et al. [20] found positive changes in motor function, walking speed, and balancing ability after 3-month participation in Tai Chi Yuttari exercise classes and suggested that Tai Chi Yuttari exercise classes are useful as a care prevention program. However, no previous study has examined whether Tai Chi Yuttari exercise is effective in extending longevity and LTC-free durations despite these outcomes being important clinical endpoints.

Thus, the purpose of this survey was to verify whether participation in Tai Chi Yuttari exercise is associated with the medium- to long-term delay in the death of participants and new certification for LTC in Kitakata City. The results of this study will provide information for the widespread use of Tai Chi Yuttari exercise to help maintain the living functions of community-dwelling older people.

## 2. Materials and Methods

### 2.1. Design

This study employed a historical cohort design to analyze data obtained from a survey.

### 2.2. Participants, Setting, and Sampling

Kitakata City in Fukushima Prefecture, Japan, the site of this survey, has a population of approximately 45,000. In March 2003, the city was the first in Japan to declare itself a “Tai Chi City” and has been conducting Tai Chi Yuttari exercise classes throughout the city. For this survey, we used (1) data from the Basic Resident Register of Kitakata City, (2) data from Kitakata City’s LTC applications, and (3) data from participants in the Tai Chi classroom, which is a project of Kitakata City. First, from the Basic Resident Register data, all those who participated in Tai Chi classes at least once in the fiscal years 2011–2015 (participation group) were extracted. Next, control individuals were extracted from those who had never participated in these classes in the fiscal years 2011–2015 (non-participation group). The inclusion criterion was age ≥ 65 years at the beginning of observation. However, those who received support/certification for LTC at baseline were excluded because it was considered that there was a strong association between death and new certification for LTC. Two individuals in the non-participation group per one individual in the participation group were extracted from the Basic Resident Register data with age-, sex-, and residential area-matched data to ensure no significant differences between the groups with respect to the distributions of these variables. A total of 128 participants and 256 non-participants were extracted. Of these, 23 individuals in the participation group and 54 individuals in the non-participation group, who were certified as requiring support/care at baseline, were excluded. Thus, 105 participants in the participation group and 202 individuals in the non-participation group were included in the analysis.

### 2.3. LTC System in Japan

All Japanese citizens aged ≥65 years have been insured through the national public LTC service since 2000. To assess older people’s need for the national public LTC service, municipal governments conduct interviews and surveys on concerns about their living conditions. The level of required LTC is determined by the Care Needs Certification Board, which comprises medical professionals and other specialists. Certification is divided into eight categories: not certified, support levels 1 and 2, and care levels 1–5. Individuals certified at a care level need more care than those at a support level, and higher grades indicate the necessity for more support or care. Insurance benefits are provided and are available for a variety of LTC insurance services such as nursing home visits and short-term admission to a care facility. The upper limit of insured benefits is determined according to the categories of certification. Certification has a term of validity and an individual’s category is regularly revised, similar to the first application. In addition, insured individuals may apply to a municipality for a change in category certification whenever the degree of necessity of care changes (e.g., functional or cognitive decline in the process of aging or sudden hospitalization) from that at application time.

### 2.4. Tai Chi Yuttari Exercise

Tai Chi Yuttari exercise includes two types of activities that participants perform in a sitting or standing position. In the sitting position, participants sit on the edge of a chair without touching the backrest and flex their knee joint to 90° with their feet flat on the floor. In the standing position, a chair or support is placed in front of participants to prevent falling. The exercise involves slow movements and does not require quick motions. In addition, the exercise is simple enough to be learned by watching it on a DVD [21]. Tai Chi Yuttari exercise was developed by a professional team to incorporate the multiple benefits of Tai Chi exercises [22]. A Tai Chi Yuttari exercise class is a sixty-minute program. Staff members measure participants’ blood pressure and check their physical condition so that participants can exercise safely. Participants do warming-up (gentle movement of bodily joints of an extremity) and cooling-down exercises (relaxation of the whole body and deep breathing) for the opening and last 5 min. Participants do Tai Chi exercises in a sitting position for 25 min. Following 5 min of rest, they are divided into 2 groups; one group is for participants who can do exercise in a standing position, and the other one is for participants who can do exercise only in a sitting position. They do Tai Chi exercises for 20 min in a standing or sitting position according to their functional status.

### 2.5. Data Collection

The analysis data were obtained from the Kitakata City Office. Each individuals’ age, sex, residential area (comprising seven regions within Kitakata City), and certification records for LTC (date of certification and certified category, if applicable) were collected for both the participation and non-participation groups. In addition, the status of participation in Tai Chi Yuttari exercise classes, that is, whether or not each individual participated in the classes in each fiscal year (2011–2015), was collected for the participation group.

### 2.6. Observation Period

The observation start date (baseline) was April 1 of the year in which the participant first participated in the Tai Chi Yuttari exercise class for the participants in the participation group. The observation start date for those in the non-participant group was matched to that of their counterparts in the participation group.

### 2.7. Endpoints

Two endpoints were set to evaluate the effect of participation in the Tai Chi Yuttari exercise classes: (1) death or (2) new certification for the need of LTC. The number of days from the observation start date to each individual’s (1) date of death or (2) date of certification as needing LTC was calculated. The observation end date was set to 5 October 2021, and observation was terminated on this date for those who survived until this date or were not certified as needing LTC.

### 2.8. Statistical Analyses

The association between participation in Tai Chi Yuttari exercise classes and longevity was analyzed using the Kaplan–Meier method. The log-rank test was used to assess the statistical significance of the difference in the survival curves. Analysis with sex stratification was also performed to examine the different effects of participation in Tai Chi Yuttari exercise classes and outcomes between men and women. The level of significance for all analyses was set at *p* < 0.05. All data were analyzed using SPSS Statistics for Windows, version 21 (IBM, Armonk, NY, USA).

## 3. Results

A total of 105 and 202 participants in the participation and non-participation groups, respectively, were observed. No significant differences were observed in age or sex distribution between the groups (Table 1). 

### 3.1. Association between Participation in Tai Chi Yuttari Exercise Classes and Longevity

**Figure 1:**
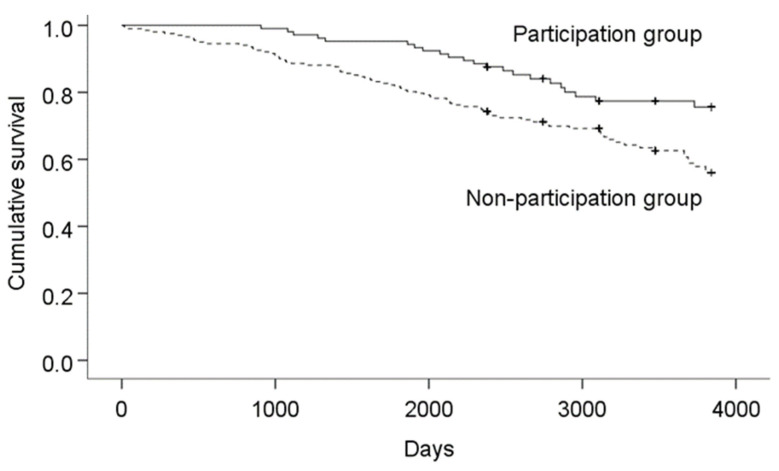
Comparison of survival duration between the participation and non-participation groups.

The number of survivors at the end of the observation was 83 out of 105 (79.0%) in the participation group and 127 out of 202 (62.9%) in the non-participation group. The numbers of days from the start of observation to death (or the end of observation) in the participation and non-participation groups (Mean Standard Error) were 3080 (779) days and 2760 (1056) days, respectively. The survival duration was longer in the participation group than in the non-participation group (χ^2^ = 8.782, *p* = 0.003; Figure 1). Regarding the results of stratified analysis by sex, out of all individuals followed, 16 men (15.2%) in the participation group and 35 men (17.3%) in the non-participation group were observed. At the end of the observation, 12 out of 16 (75.0%) and 21 out of 35 (60.0%) were alive in the participation and non-participation groups, respectively. The numbers of days from the start of observation to death (or the end of observation) in the participation and non-participation groups were 3316 (234) days and 2228 (204) days, respectively. The survival duration was longer in the participation group than in the non-participation group (χ^2^ = 7.875, *p* = 0.005). Regarding women, of all individuals followed, 89 (84.8%) in the participation group and 167 (82.7%) in the non-participation group were observed. At the end of the observation, 71 out of 89 (79.8%) and 116 out of 167 (69.5%) were alive in the participation and non-participation groups, respectively. The numbers of days from the start of observation to death (or the end of observation) in the participation and non-participation groups were 3499 (78) days and 3247 (82) days, respectively. The difference in survival duration between the participation and non-participation groups was borderline significant (χ^2^ = 3.396, *p* = 0.065).

### 3.2. Association between Participation in Tai Chi Yuttari Exercise Classes and Durations until LTC Certification

**Figure 2:**
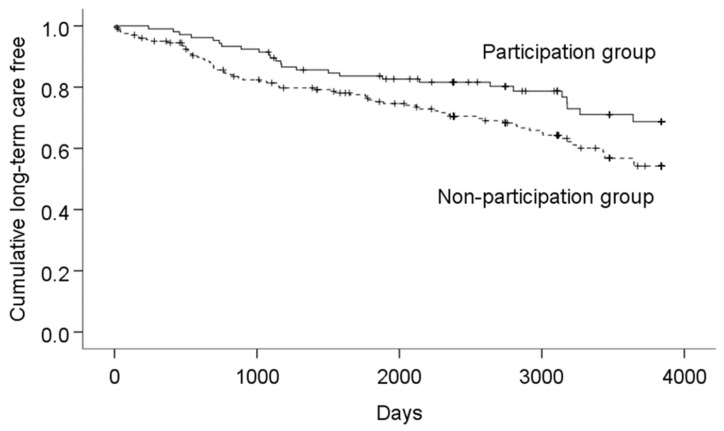
Comparison of days until receiving a new long-term care certification between the participation and non-participation groups.

The proportions of those who were not certified as requiring LTC at the end of the observation were 79 out of 105 (75.2%) in the participation group and 130 out of 202 (64.4%) in the non-participation group. The numbers of days from the start of observation to receiving LTC certification (or the end of observation) in the participation and non-participation groups were 2703 (1047) days and 2304 (1267) days, respectively. The duration was longer in the participation group than in the non-participation group (χ^2^ = 5.354, *p* = 0.021; Figure 2). Regarding the results of stratified analysis by sex, for men, at the end of the observation, 12 out of 16 (75.0%) and 21 out of 35 (60.0%) were not certified as requiring LTC in the participation and non-participation groups, respectively. The numbers of days from the start of observation to receiving LTC certification (or the end of observation) in the participation and non-participation groups were 3149 (303) days and 2541 (262) days, respectively. No significant difference in duration without LTC certification was observed between the participation and non-participation groups (χ^2^ = 1.909, *p* = 0.167). For women, at the end of the observation, 67 out of 89 (75.3%) and 109 out of 167 (65.3%) were not certified as requiring LTC in the participation and non-participation groups, respectively. The numbers of days from the start of observation to receiving LTC certification (or the end of observation) in the participation and non-participation groups were 3256 (117) days and 2956 (103) days, respectively. The difference in duration without LTC certification between the participation and non-participation groups was borderline significant (χ^2^ = 3.636, *p* = 0.057).

## 4. Discussion

The results of this study showed that those who participated in Tai Chi Yuttari exercise classes survived longer and took longer to be certified as requiring care than those who did not participate. While previous studies have already found that exercise had an enhancing effect on physical function, this study added information on the direct benefit of participating in Tai Chi Yuttari exercise classes on longevity and extending independent lives. These effects are in line with the goals of the national health promotion measures indicated by the Ministry of Health, Labour, and Welfare of Japan [23].

The benefits of Tai Chi Yuttari exercise, other than improved functional ability, could contribute to the association between longevity and participation in Tai Chi Yuttari exercise classes. Yamamoto et al. [24] reported that Tai Chi Yuttari exercise had effects (such as a decrease in oxidative stress and maintaining antioxidant capacity) similar to those after comfortable walking in older women. Antioxidant action maintains the oxidant/antioxidant balance in the body with respect to oxidative stress [25]. Regular physical activity reduces oxidative stress and increases antioxidant capacity, particularly in post-menopausal women [26]. Thus, participating in Tai Chi Yuttari exercise classes might be as useful in maintaining metabolic function as walking. The production of oxidants during ordinary metabolism damages DNA, proteins, and fat, causing cardiovascular disease, diabetes, immune system decline, and brain dysfunction [27]. Aging has been reported to increase oxidative stress and decrease antioxidant capacity [28]. It might be possible that Tai Chi Yuttari exercise is effective in maintaining metabolic function, which generally decreases with advancing age, and it could contribute to reduced risk of some diseases that lead to death.

Mori et al. [29] reported that Tai Chi Yuttari exercise also improves vascular function. A previous study showed that exercise habits could suppress the age-related decrease in central arterial extensibility [30]. A longitudinal study reported that arterial extensibility was improved after a relatively short period of oxygenated exercise of approximately 2–6 months [31,32]. Previous studies reported the effects of Tai Chi as an aerobic exercise. Tai Chi not only improves balance and fall prevention but also improves the maximum oxygen intake in patients after coronary artery bypass graft surgery [33] and increases exercise tolerance in patients with heart failure [34]. The improvement in arterial extensibility observed after Tai Chi with relaxed exercise may reflect the characteristics of such aerobic exercise. In summary, Tai Chi Yuttari exercise may improve bodily function when designed properly and subsequently contribute to longevity.

A scoping review reported that risk factors for LTC certification included physical function [35]. Previous studies suggested that the benefits of Tai Chi Yuttari exercise included improvements in physical function such as improvement in balance ability (evaluated by standing on one leg) and flexibility (evaluated by bending forward) [22]. In a previous study by Fujimoto et al. [20], which examined the association between the implementation of Tai Chi Yuttari exercise and new certification for LTC, Tai Chi Yuttari exercise improved the muscle strength and balance function of the lower limbs. The improvement in walking ability reduced the risk of falls, leading to a reduction in the risk of new certification for LTC, which supports the results of the present study. To reduce the percentage of people certified as requiring LTC in the city, it is suggested that the functional ability and risk of falls were improved through participation in Tai Chi Yuttari exercise classes, which subsequently contributed to a decreased risk of falls and LTC certification. As a result of these direct and indirect effects, the percentage of people certified as requiring LTC can be reduced.

Furthermore, social participation through Tai Chi exercise classes may be associated with a decreased risk of the need for LTC and mortality among elderly patients [36]. The mechanisms regarding why and how social participation affects healthy aging and death are not yet known, but growing evidence suggests that social participation stimulates the body and brain, helping participants remain highly functional [37]. Another study suggested that individuals who engage in social participation may have easier access to social support [38]. With regard to biomedical mechanisms, social participation may suppress inflammatory markers and reduce physical stress [39].

Interestingly, we observed a sex difference in the association between participation in Tai Chi Yuttari exercise and outcomes; participation was associated with survival duration in men only. Previous studies examining sex differences in the effects of exercise on NCDs are controversial [40]. The Framingham study indicated that moderate or intense physical activity is protective against stroke in men but not in women [41]. Another study examining the effects of exercise intervention found that male participants showed a greater loss in body weight compared to sedentary controls, but a similar effect was not observed among female participants [42]. It is possible that the effect of exercise on body composition and risk of specific diseases differs between men and women and could explain the sex difference observed in the present study regarding the association between participation in Tai Chi Yuttari exercise and prolonged survival durations. 

In Japan, after the implementation of the LTCI system in 2000, the number of people certified for LTCI service rapidly increased, with a corresponding rise in the financial burden on the government. Therefore, the government initiated a disability prevention program and a high-risk approach with appropriate prevention programs in each community [43]. Tai Chi Yuttari exercise could fit this national policy, as it has low risk for adverse effects. Furthermore, the World Health Organization has developed an Age-friendly Cities Framework [44], which contains eight domains that can help to identify and address barriers to the well-being and social participation of older people. Of these interconnected domains, Tai Chi Yuttari exercise can contribute to the achievement of “community and healthcare” and “social participation.” Tai Chi is regarded as a community-based group exercise that can ensure active aging and subsequent better quality of life [45].

The strengths of this study are that the effects of Tai Chi Yuttari exercise could be compared in age- and sex-matched individuals between the participation and non-participation groups to reduce potential selection bias. In addition, in this study, all those who participated in Tai Chi Yuttari exercise classes during the defined period were examined at baseline, which enabled the generalization of the results of this study. On the other hand, there are four limitations to note when interpreting the results of this survey. First, the causation between participation in Tai Chi Yuttari exercise classes and outcomes could not be confirmed due to the nature of the retrospective cohort design. However, it is reasonable to assume that participation in Tai Chi Yuttari exercise classes was effective in improving outcomes. Second, this study used death as its endpoint and did not assess items related to healthy life expectancies, such as a decline in physical and mental function leading up to death or a decline in independence. In addition, participants’ comorbidity and lifestyle habits were also not collected, although they are thought to affect longevity directly or indirectly [14,46,47]. Nevertheless, the presence or absence of comorbidity could be partly controlled by excluding individuals with LTC certification at baseline. A meta-analysis of the dose–response relationship between non-vigorous physical activity and all-cause mortality found the largest benefit in individuals who moved from no activity to low levels of activity [48]. Regarding the length of exercise duration, a significant reduction in the number of falls and fall-associated injuries, improved physical function, and enhanced cognition were observed independent of exercise frequency [49]. Thus, although it would be better to have detailed information on the domains of exercise or frequency, participation in Tai Chi Yuttari exercise seemed to explain the effect of delayed death and LTC certification to some extent. In addition, nutrition is a well-known factor associated with frailty in older adults, which could be more closely associated with LTC certification [50]. Education has also been reported to be associated with mortality [48]. Although we did not collect information on each participant’s education history, we enrolled participants from one municipality, and both the participation and non-participation groups were age- and sex-matched. Therefore, a drastic difference in education history between the two groups is not expected. Third, this study used the presence or absence of participation in Tai Chi Yuttari exercise classes during the observation period as a factor and did not evaluate conditions such as whether participation was interrupted or the participation rate, which may have affected the results. Finally, the sample size of this study is not large even though all the participants in Tai Chi Yuttari exercise during certain periods were included, which might cause an underestimation of an α error. Despite these limitations, this study clarified that Tai Chi Yuttari exercise might be effective in elongating longevity and the duration of independence in older people.

Tai Chi Yuttari exercise in a community setting might be beneficial for longer longevity and healthy life expectancy. In addition, the findings of this study may also be applicable in other municipalities or communities besides Kitakata City. Tai Chi Yuttari exercise is advantageous in that even frail older people can participate safely and easily and it can be implemented in communities with large proportions of frail older people. Therefore, it is desirable that Tai Chi Yuttari exercise is disseminated in more municipalities, and local instructors can be beneficial for dissemination. 

Furthermore, as this study found that participation was associated with delayed death in men only, it is desirable that more male older residents participate in Tai Chi Yuttari exercise classes. However, as this study showed, a larger proportion of participants were women, which is consistent with a previous study suggesting that women are more likely to participate in sports clubs compared with men [51]. This variance in sex distribution might be due to differences in attitude or motivation for participation in healthcare services in general. A previous study suggested that women use more medical services because they are more sensitive to disease symptoms and are interested in health or experience more morbidities than men [52]. In fact, in preventive care events held in Japan, women constitute the majority population. In the future, we need to consider any intervention that can encourage more male older residents to participate in Tai Chi Yuttari exercise classes. 

## 5. Conclusions

Tai Chi Yuttari exercise might be effective in the medium to long term in delaying the death of participants and new certification for LTC in Kitakata City. As participation in Tai Chi Yuttari exercise might be effective in prolonging longevity in men, the participation of more men should be encouraged for better overall health benefits for older people.

## Figures and Tables

**Table 1:** Demographic characteristics between the participation and non-participation groups.

	Participation Group *n* = 105	Non-Participation Group *n* = 202	*p*
Age, mean ± SD	78.8 ± 6.7	78.7 ± 6.5	0.888
Sex, female, n (%)	89 (84.8%)	167 (82.7%)	0.641

SD, standard deviation.

## Data Availability

Due to the nature of this research, participants in this study did not agree for their data to be shared publicly, so supporting data are not available.
